# Effect of CUX1 on the Proliferation of Hu Sheep Dermal Papilla Cells and on the Wnt/β-Catenin Signaling Pathway

**DOI:** 10.3390/genes14020423

**Published:** 2023-02-07

**Authors:** Hui Zhou, Sainan Huang, Xiaoyang Lv, Shanhe Wang, Xiukai Cao, Zehu Yuan, Tesfaye Getachew, Joram M. Mwacharo, Aynalem Haile, Kai Quan, Yutao Li, Antonio Reverter, Wei Sun

**Affiliations:** 1 College of Animal Science and Technology, Yangzhou University, Yangzhou 225009, China; 2 International Joint Reserarch Laboratory in Universities of Jiangsu Province of China for Domestic Animal Germplasm Resources and Gentic Improvement, Yangzhou University, Yangzhou 225009, China; 3 Joint International Research Laboratory of Agriculture and Agri-Product Safety of Ministry of Education of China, Yangzhou University, Yangzhou 225009, China; 4 International Centre for Agricultural Research in the Dry Areas, Addis Ababa 999047, Ethiopia; 5 College of Animal Science and Technology, Henan University of Animal Husbandry and Economics, Zhengzhou 450046, China; 6 CSIRO Agriculture and Food, 306 Carmody Rd, St Lucia, QLD 4067, Australia; 7 “Innovative China” “Belt and Road” International Agricultural Technology Innovation Institute for Evaluation, Protection, Improvement on Sheep Genetic Resource, Yangzhou 225009, China

**Keywords:** CUT-like homeobox 1 protein, Hu sheep, dermal papilla cells, Wnt/β-catenin

## Abstract

CUT-like homeobox 1 protein (CUX1), also called CUX, CUTL1, and CDP, is a member of the DNA-binding protein homology family. Studies have shown that *CUX1* is a transcription factor that plays an important role in the growth and development of hair follicles. The aim of this study was to investigate the effect of *CUX1* on the proliferation of Hu sheep dermal papilla cells (DPCs) to reveal the role of *CUX1* in hair follicle growth and development. First, the coding sequence (CDS) of *CUX1* was amplified by PCR, and then *CUX1* was overexpressed and knocked down in DPCs. A Cell Counting Kit-8 (CCK8), 5-ethynyl-2-deoxyuridine (EdU), and cell cycle assays were used to detect the changes in the proliferation and cell cycle of DPCs. Finally, the effects of overexpression and knockdown of *CUX1* in DPCs on the expression of *WNT10*, *MMP7*, *C-JUN*, and other key genes in the Wnt/β-catenin signaling pathway were detected by RT-qPCR. The results showed that the 2034-bp CDS of *CUX1* was successfully amplified. Overexpression of *CUX1* enhanced the proliferative state of DPCs, significantly increased the number of S-phase cells, and decreased the number of G0/G1-phase cells (*p* < 0.05). *CUX1* knockdown had the opposite effects. It was found that the expression of *MMP7*, *CCND1* (both *p* < 0.05), *PPARD*, and *FOSL1* (both *p* < 0.01) increased significantly after overexpression of *CUX1* in DPCs, while the expression of *CTNNB1* (*p* < 0.05), *C-JUN*, *PPARD*, *CCND1*, and *FOSL1* (all *p* < 0.01) decreased significantly. In conclusion, *CUX1* promotes proliferation of DPCs and affects the expression of key genes of the Wnt/β-catenin signaling pathway. The present study provides a theoretical basis to elucidate the mechanism underlying hair follicle development and lambskin curl pattern formation in Hu sheep.

## 1. Introduction

There are great differences in the quality of the lambskin of Hu sheep. According to the width of the pattern, it can be classified into four types: small waves (pattern width 0.5–1.25 cm), medium waves (pattern width 1.25–2 cm), large waves (pattern width 2 cm or more), and straight hair (no pattern). Wavy lambskin with small waves is the most desired type, and the straight hair pattern is the least desired [1]. Curvature of wool is one of the key factors in determining the quality of lambskin. Dermal papilla cells (DPCs) are the regulatory center of hair follicle growth and development, and the proliferation of DPCs plays an important role in hair follicle growth and wool curvature. Therefore, studying the regulatory mechanism of the proliferation of DPCs is beneficial to reveal the molecular mechanism underlying pattern formation in lambskin.

The hair follicle is an important accessory organ of the skin and includes both dermal and epidermal parts. The dermis contains the outer root sheath and the inner root sheath, while the epidermis includes the hair papilla and the dermal sheath. DPCs are located in the dermis of the hair follicle and are a special type of interstitial cells that exist at the base of the hair follicle, where they are surrounded by hair matrix cells, and play an important regulatory role in the formation, growth, and circulation of hair [2]. Rompolas et al. [3] found that, after elimination of the hair papilla cells by laser, the hair follicle will not enter the anagen phase and the shape of the hair will change, which indicates that the number of DPCs in the hair follicle can determine the curvature of the hair. Chi et al. [4] showed that, in mice, four different hair types can be produced in a hair follicle growth cycle, including guard hair (guard), tapered hair (awl), angular hair (auchene), and zigzag hair (zigzag), and differences in dermal papilla shape and the number of DPCs in it are observed to be directly related to the type of hair, with a reduction in the number of DPCs resulting in a significant reduction in the length and thickness of the hair. Furthermore, it has been suggested that the main cause of hair follicle curvature is the formation of multiple papillary centers in the dermal papillae that can function autonomously, which likely causes asymmetric hair growth and curvature [5]. The abovementioned studies have shown the important role of hair papilla cells in influencing hair follicle growth and development, and hair curvature. Therefore, we studied the mechanisms by which DPCs affect lambskin quality.

The homology frame CUT-like homeobox 1 protein (CUX1), also called CUX, CUTL1, and CDP, is a member of the homology domain family of DNA-binding proteins. Previous studies have shown that inactivation of *CUX1* in mice leads to many abnormal manifestations, such as growth retardation, whisker curling, altered hair follicle morphology, and male sterility [6,7]. Although there is no study directly indicating that *CUX1* affects the growth and development of DPCs, previous research has found that miR-143 is a differentially expressed miRNA that may affect hair follicle development in Hu sheep [8]. It has been found that miR-143 affects the expression of key genes in the Wnt/β-catenin signaling pathway by regulating the target genes *CUX1* and *KRT71*, thereby inhibiting the proliferation of DPCs. Therefore, we hypothesized that *CUX1* may also directly affect the proliferation of DPCs and the curvature of hair growth.

Numerous studies have shown that many molecular signaling pathways are involved in the regulation of hair follicle growth and development, such as the Wnt/β-catenin signaling pathway, the BMP signaling pathway, the TGF-β signaling pathway, and the Notch signaling pathway [9,10,11,12,13]. The classical Wnt/β-catenin signaling pathway plays a positive role in hair folliculogenesis and growth [14]. It has been found that *Wnt10b* regulates the growth cycle of hair follicles and is mainly expressed during the anagen phase of hair follicles and is not expressed during the regression and resting phases. During hair follicle growth, high expression of *Wnt10b* has been detected in the bulge of the hair follicle, while Wnt10b protein expression has been detected near the hair papilla cells. When *Wnt10b* is highly expressed, it inhibits the activation of downstream β-catenin in the Wnt/β-catenin signaling pathway [15,16]. Therefore, it has been suggested that *Wnt10b* inhibits the activation of the Wnt/β-catenin signaling pathway and allows hair follicles in the resting phase to enter the anagen phase.

In this study, we aimed to investigate the role of *CUX1* in DPCs and whether *CUX1* can regulate the Wnt/β-catenin pathway, to provide a theoretical basis to elucidate the mechanisms underlying lambskin pattern formation in lambs.

## 2. Materials and Methods

### 2.1. Experimental Animals

Healthy and disease-free 3-day-old lambs (Jiangsu Xuzhou Su Sheep Industry Co.) were selected for this experiment. Three pairs of full sibling individuals were divided into small waves groups and straight wool groups. Scapular skin tissue samples (1 cm^2^), as well as heart, liver, spleen, lung, kidney, and muscle tissue samples, were taken in triplicate, snap-frozen in liquid nitrogen, and stored at −80 °C until use.

DPCs were isolated and cultured from Hu sheep skin in our laboratory. Total RNA of DPCs was extracted using TRIzol (TIANGEN, Beijing, China) and stored at −80 °C. The first strand of cDNA was synthesized by FastKing gDNA Dispelling RT SuperMix (TIANGEN, Beijing, China), and stored at −80 °C.

Our experimental protocol was approved by the Animal Ethics Committee of Yangzhou University (NXFC2020-NF-1).

### 2.2. Cell Culture and Cell Transfection

DPCs were cultured in DMEM/F12 containing 5% fetal bovine serum and 1% penicillin–streptomycin at 37 °C with 5% CO_2_. DPCs in the growth stage were inoculated uniformly in 6-well, 12-well, and 96-well plates, and when the cells reached 50–80% confluency, they were transfected with jetPRIME reagent following the manufacturer’s instructions. RNA and protein were extracted and stored at −80 °C until use.

### 2.3. Eukaryotic CUX1 Expression Vector Construction

Primers to amplify the full-length *CUX1* coding sequence (CDS) were designed using Oligo 6 software based on the *CUX1* sequence downloaded from NCBI. The full-length CDS was amplified using lamb skin tissue cDNA as a template. The fragment was cloned into the PEX-1 overexpression vector, and successful cloning was confirmed by double digestion with QuickCut™ XhoI (Takara, Japan) and QuickCut™ NotI (Takara, Japan). The overexpression vector were sent to the Tsingke Biotechnology Co., Ltd. (Nanjing, China) for verification.

The *CUX1* primer sequences are as follows:

PEX-1-CUX1-F: CCCaagcttATGGCGGCCAATGTGGGATC

PEX-1-CUX1-R: CCGctcgagACACTGCCACAGGTCGCCG

### 2.4. siRNA Synthesis

The siRNA-*CUX1* was designed and synthesized by Shanghai Jima Pharmaceutical Co., Ltd. (Shanghai, China). The sequences are shown in Table 1.

### 2.5. Cell Proliferation Assay

First, hair papilla cells were transfected with the *CUX1* overexpression vector (PEX-1-CUX1), interfering RNA (siRNA-232), and negative controls (PEX-1 and siRNA-NC). At 36 h after transfection, they were washed twice with PBS, 10 μL CCK-8 reagent (Tecan, Shanghai, China) was added to each well and the culture was continued at 37 °C for 2 h, then the OD_450_ values were measured at 0, 24, 48, and 72 h using a microplate reader (Tecan, Männedorf, Switzerland). The CCK-8 results were analyzed by GraphPad Prism 8. In addition, transfected cells were processed with the Cell-Light TM EdU Apollo 567 In Vitro Kit (RiboBio, Guangzhou, China) and photographed with an inverted fluorescence microscope (Nikon, Tokyo, Japan). The EDU pictures were analyzed by ImagePro Plus 6.0.

### 2.6. Cell Cycle Assay

Hair papilla cells were transfected, and after 48 h, cell suspensions were collected according to the Cell Cycle and Apoptosis Kit (Beyotime, Shanghai, China). Changes in DNA content were detected by flow cytometry (BD, Franklin Lakes, NJ, USA) and the results were analyzed by ModFit.

### 2.7. RT-PCR and Wnt/β-Catenin Signal Path Detection

According to the sheep gene sequences published by Gene Bank, the primers of *CUX1*, *CDK2*, *PCNA*, *CYCLIN D1*, *WNT10*, *MMP7*, *C-JUN*, *PPARD*, *CCND1*, *FOSL1*, *CTNNB1*, and *GAPDH* were designed by NCBI (https://www.ncbi.nlm.nih.gov/ (accessed on 30 January 2021)) (Table 2).

According to the TB GreenTM Premix EX Taq^TM^ (Takara, Tapan), the reaction System: 2 × TB Green *Premix Ex Taq* II 12.5 μL, F 1 μL, R 1 μL, cDNA 2 μL, RNase-Free ddH_2_O 8.5 μL.

Reaction procedure: 95 ℃ 30 s, 95 ℃ 5 s, 60 ℃ 30 s, 40 cycles.

### 2.8. Western Blot

After the DPC_S_ were transfected for 48 h, proteins were extracted with RIPA (Beyotime, Shanghai, China). Protein concentrations were determined with a BCA Protein Concentration Kit (Beyotime, Shanghai, China). After adjusting the protein concentrations with PBS, proteins were separated by SDS-PAGE in 10% gels that were prepared with the PAGE Gel Rapid Preparation Kit (Yamei, Shanghai, China). Then the proteins were transferred to PVDF membranes, which were blocked in 5% skimmed milk powder solution at room temperature for 1 h. Next, the membranes were incubated with primary antibody (Proteintech, Wuhan, China) (Table 3), washed, and incubated with secondary antibody (ABclonal, Wuhan, China) (Table 3). Protein bands were visualized with the ECL chemiluminescent substrate kit (Biosharp, Hefei, China), and images were analyzed with Image-Pro Plus 6.0 (Media Cybernetics, USA).

### 2.9. Data Analysis

SPSS 17.0 and Excel were used to analyze the experiment data. Graphs were plotted with GraphPad Prism 8. Data are represented as the mean ± standard error of the mean(SEM). The RT-PCR results used the 2^-∆∆CT^ method. The independent sample *t*-test was used to perform variance and the one-way ANOVA was used to perform variance. The value of *p* < 0.05 represented a significant difference; *p* < 0.01 represented an extremely significant difference.

## 3. Results

### 3.1. CUX1 Expression in Different Tissues

**Figure 1:**
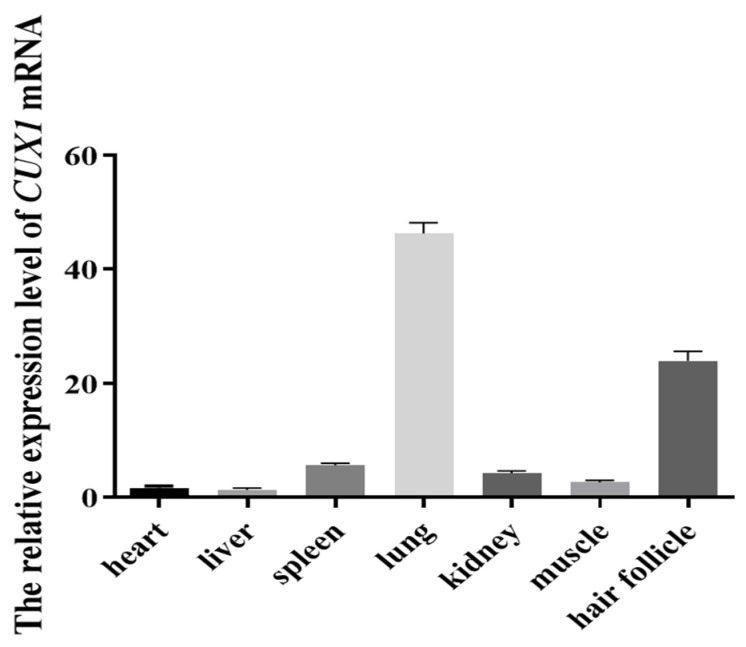
The relative expression level of *CUX1* mRNA in different tissues of Hu sheep.

The *CUX1* expression levels in heart, liver, spleen, lung, kidney, dorsal muscle, and skin samples were determined by RT-qPCR. *CUX1* expression was relatively high in the skin, suggesting that *CUX1* may be involved in the regulation of hair follicle development (Figure 1).

### 3.2. Construction of a Eukaryotic CUX1 Expression Vector

RNA was extracted from Hu sheep skin tissue and re-transcribed into cDNA. Gel electrophoresis of the PCR product resulted in a bright band appearing at 2034 bp. The recovered PCR product was inserted into a linearized PEX-1 vector, host bacteria were transformed, and after 16 h the plasmid was extracted to obtain the PEX-1-CUX1 eukaryotic overexpression vector. Successful cloning was confirmed by double digestion with KpnI and XhoI. Bright bands appeared at 4700 bp and 2034 bp, indicating that the PEX-1-CUX1 eukaryotic overexpression vector was successfully constructed.

### 3.3. Transfection of DPCs with the CUX1 Overexpression Vector

**Figure 2:**
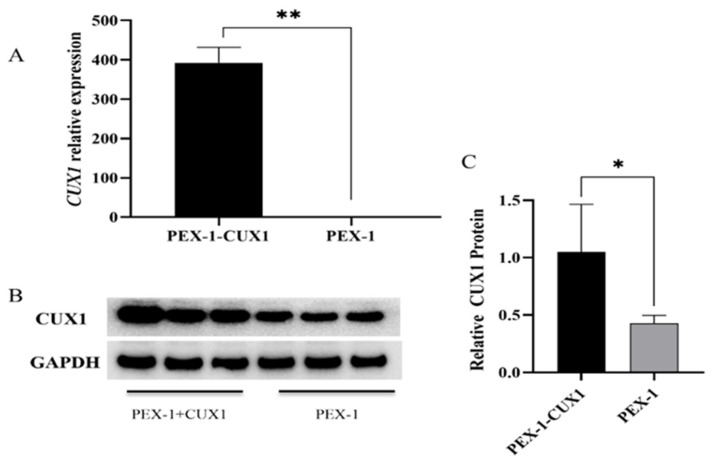
mRNA and protein expression levels of *CUX1* in Hu sheep DPCs after *CUX1* overexpression. (**A**)The relative expression levels of *CUX1* mRNA. (**B**)The relative expression levels of CUX1 protein. (**C**) The relative expression levels of CUX1 protein were analyzed by Image Lab. (*) stands for *p* < 0.05, (**) stands for *p* < 0.01.

DPCs were transfected with PEX-1-CUX1, and the mRNA and protein expression levels of *CUX1* were detected by RT-qPCR and Western blot (WB), respectively. *CUX1* mRNA expression (*p* < 0.01) (Figure 2A) and CUX1 protein expression (*p* < 0.05) (Figure 2B,C) were significantly increased, confirming that the plasmid could be used in subsequent experiments.

### 3.4. CUX1 Knockdown in DPCs

**Figure 3:**
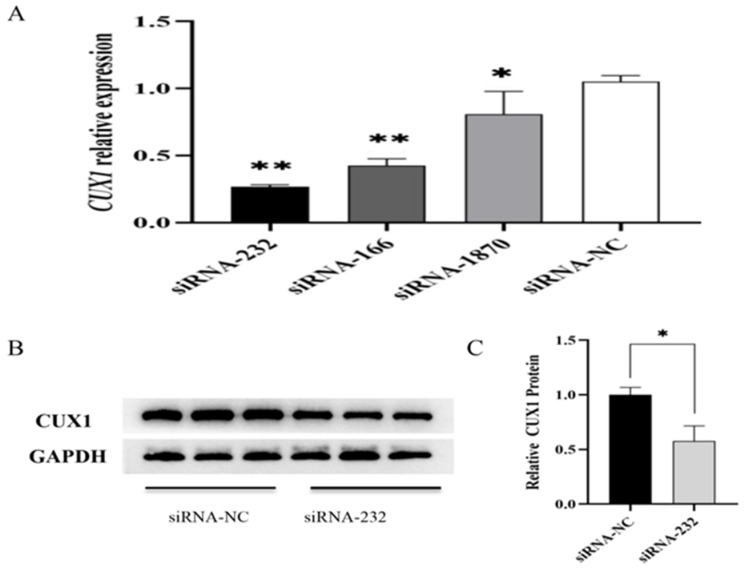
*CUX1* mRNA and protein expression levels in Hu sheep DPCs after knockdown by siRNA-232. (**A**) The relative expression levels of *CUX1* mRNA. (**B**) The relative expression levels of CUX1 protein. (**C**) The relative expression levels of CUX1 protein were analyzed using Image Lab. * *p <* 0.05, ** *p* < 0.01.

DPCs were transfected with three different *CUX1* knockdown fragments. *CUX1* mRNA levels were determined by RT-qPCR. The siRNA-232 group showed the strongest reduction in *CUX1* mRNA expression (Figure 3A). Next, CUX1 protein expression in the siRNA-232 group was determined by WB. It was found that siRNA-232 significantly downregulated the protein expression of CUX1 (*p* < 0.05) (Figure 3B,C). Based on these results, siRNA-232 was selected for subsequent experiments.

### 3.5. CUX1 Promotes the Proliferation of DPCs

**Figure 4:**
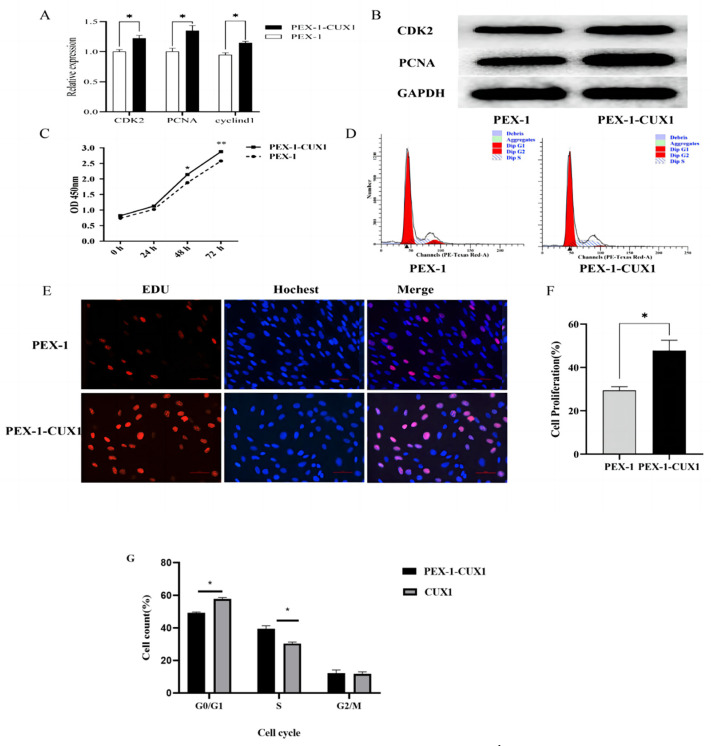
Overexpression of *CUX1* promoted proliferation of Hu sheep DPCs. (**A**) mRNA expression levels of *PCNA*, *CYCLIN D1*, and *CDK2* after *CUX1* overexpression. (**B**) The relative expression levels of CDK2 and PCNA protein after *CUX1* overexpression. (**C**) OD_450_ values in the CCK8 assay after *CUX1* overexpression. (**D**) Cell-cycle analysis of Hu sheep DPCs overexpressing *CUX1* by flow cytometry. (**E**) The number of proliferating Hu sheep DPCs after *CUX1* overexpression was detected by the EdU assay. EdU staining (red) indicates proliferating cells; Hoechst staining (blue) indicates nuclei. (**F**) The proportion of EdU-positive Hu sheep DPCs. (**G**) The rate of different periods of the cell cycle * *p* < 0.05, ** *p* < 0.01.

We examined the effects of *CUX1* overexpression on the proliferation of DPCs. When *CUX1* was overexpressed, the mRNA expression levels of the cell proliferation genes *CDK2*, *PCNA*, and *CYCLIN D1* were increased(Figure 4A), and the protein expression levels of CDK2 and PCNA were upregulated (Figure 4B). The CCK-8 assay was used to detect DPC proliferation. At 48 h after transfection with the *CUX1* overexpression plasmid, the proliferation rate of DPCs was significantly higher than that of the control group (*p* < 0.05), and the highest proliferation rate was reached at 72 h (*p* < 0.01) (Figure 4C). The changes in the cell cycle of DPCs were detected by flow cytometry; it was found that after overexpression of *CUX1*, the number of cells in the S phase was significantly higher than that in the control group, and the number of cells in the G0/G1 stage was significantly reduced (Figure 4D,G). After overexpression of *CUX1*, the number of EdU-positive cells was significantly increased (*p* < 0.05) (Figure 4E,F). These results show that *CUX1* overexpression promotes the proliferation of DPCs.

**Figure 5:**
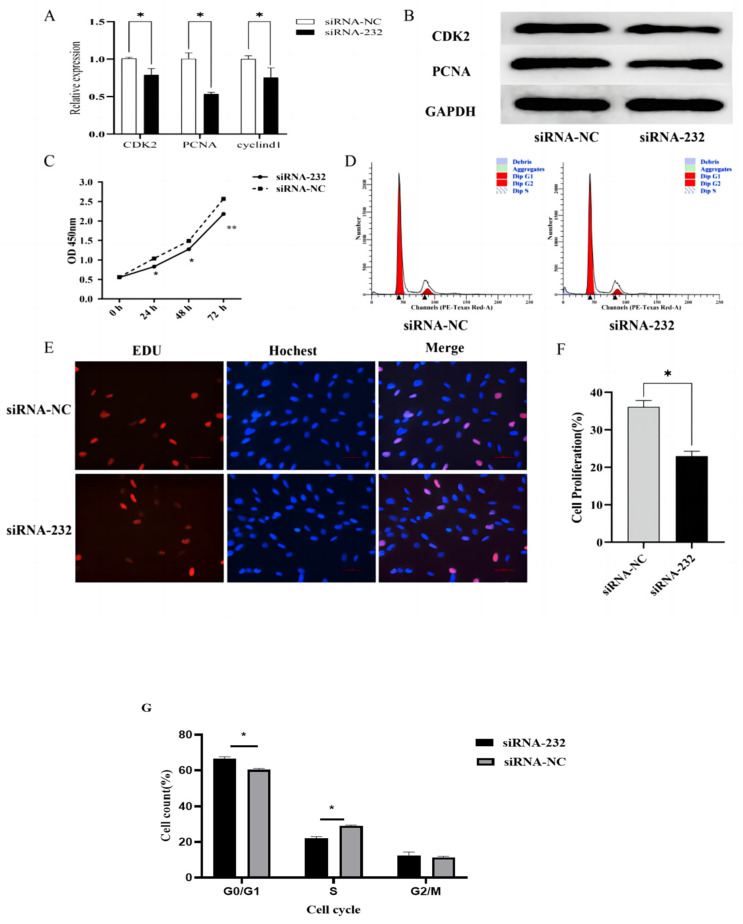
*CUX1* knockdown inhibits proliferation of Hu sheep DPCs. (**A**) mRNA expression levels of *PCNA*, *CYCLIN D1*, and *CDK2* after *CUX1* knockdown. (**B**) The relative expression levels of CDK2 and PCNA protein after *CUX1* knockdown. (**C**) OD_450_ values in the CCK8 assay after *CUX1* knockdown. (**D**,**G**) Cell-cycle analysis of CUX1-silenced Hu sheep DPCs by flow cytometry. (**E**) The number of proliferating Hu sheep DPCs after *CUX1* knockdown was detected by the EdU assay. EdU staining (red) indicates proliferating cells; Hoechst staining (blue) indicates nuclei. (**F**) The proportion of EdU-positive Hu sheep DPCs. (**G**) The rate of different periods of the cell cycle * *p* < 0.05, ** *p* < 0.01.

In addition, DPCs were transfected with the *CUX1* knockdown plasmid. After *CUX1* knockdown, the mRNA expression levels of *CDK2*, *PCNA*, and *CYCLIN D1* were significantly decreased (*p* < 0.05) (Figure 5A), and the protein expression levels of CDK2 and PCNA were also significantly reduced (Figure 5B). The CCK-8 assay showed that at 24 and 48 h after transfection, the cell proliferation rate was significantly lower than that of the control group (*p* < 0.05). This difference was highly significant at 72 h after transfection (Figure 5C). The changes in the cell cycle were detected by flow cytometry, and it was found that, after *CUX1* knockdown, the number of cells in the S phase was significantly lower than that in the control group, and the number of cells in the G0/G1 stage was significantly elevated (Figure 5D,G). The number of EdU-positive cells was significantly reduced after *CUX1* knockdown (*p* < 0.05) (Figure 5E,F). These results show that *CUX1* knockdown can inhibit the proliferation of DPCs.

### 3.6. CUX1 Regulated the Key Genes in the Wnt/β-Catenin Signaling Pathway

**Figure 6:**
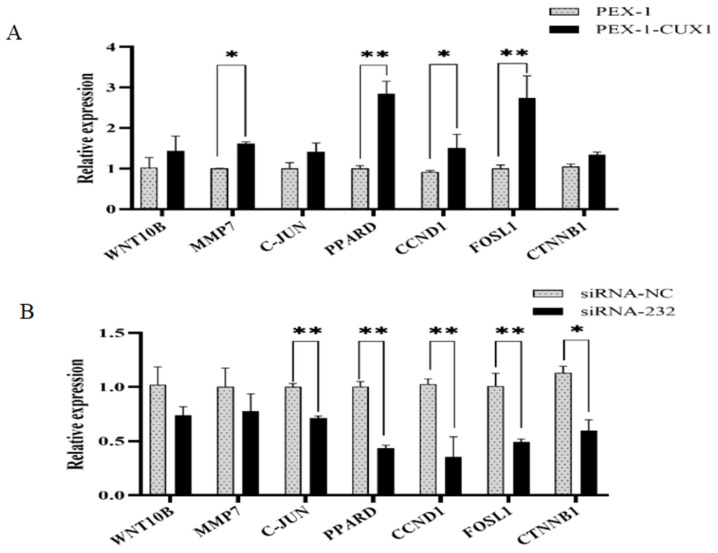
Expression of Wnt/β-catenin signaling pathway genes after *CUX1* overexpression or knockdown. (**A**) mRNA expression levels of different genes after *CUX1* overexpression. (**B**) mRNA expression levels of different genes after *CUX1* knockdown. * *p* < 0.05, ** *p* < 0.01.

To analyze the effects of *CUX1* on the Wnt/β-catenin signaling pathway, the expression of key genes in the Wnt/β-catenin signaling pathway (*WNT10*, *MMP7*, *C-JUN*, *PPARD*, *CCND1*, *FOSL1*, and *CTNNB1*) after *CUX1* overexpression and knockdown was determined by RT-PCR. *CUX1* overexpression significantly increased the mRNA expression levels of *MMP7*, *CCND1* (both *p* < 0.05), *PPARD*, and *FOSL1* (both *p* < 0.01) (Figure 6A). After *CUX1* knockdown, mRNA expression levels of *CTNNB1* were significantly increased (*p* < 0.05) and mRNA expression levels of *C-JUN*, *PPARD*, *CCND1*, and *FOSL1* were significantly decreased (*p* < 0.01) (Figure 6B). The mRNA expression levels of *WNT10* and *MMP7* were decreased, but this effect was not significant. These results suggest that *CUX1* can regulated the expression of key genes in the Wnt/β-catenin signaling pathway.

## 4. Discussion

Hu sheep are a unique white lamb breed in China, known for producing lamb skins with wavy patterns. In recent years, due to the emphasis on meat quality and reproductive performance, as well as the introduction of large quantities of foreign bloodlines in the production area, the wavy pattern characteristic of lamb skins has gradually disappeared and the quality of lamb skins has gradually deteriorated. The curvature of wool is a key factor in determining the quality of Hu sheep lamb skins, yet the molecular regulatory mechanisms determining wool curvature are not clear. Therefore, it is important to study the molecular mechanisms regulating wool bending for the conservation of lamb skin germplasm resources of Hu sheep.

DPCs are the regulatory center of hair follicle growth and development, and the number and type of DPCs are decisive for follicle growth, wool type, and wool curvature. It has been suggested that the main cause of hair follicle curvature is the autonomous functioning of multiple papilla centers formed in the dermal papillae, which likely contributes to asymmetric hair growth curvature. It has also been suggested that curly hairs originate from a hair bulb surrounded by a large number of proliferating cells, which cause curved hair growth due to the uneven distribution of their proliferation space. Since DPCs have great potential to proliferate and differentiate and are present in the center of the hair bulb, we investigated the causes of curly growth in wool from the perspective of hair papilla cell proliferation.

*CUX1* belongs to a family of homologous structural domain transcription factors that are widely expressed in a variety of tissues and have important roles in organ development, cell proliferation, and differentiation [17,18]. Studies have shown that *CUX1* can be directly regulated as a target gene by miRNAs that are involved in the regulation of cell proliferation and differentiation [8,19,20,21]. For example, miR-155 can directly downregulate *CUX1* expression and participate in the developmental process of macrophage differentiation [22]; miR-122 can inhibit mouse hepatocyte proliferation and promote differentiation by knocking down *CUX1* [23]; and miR-208a downregulates *CUX1* to regulate cardiomyocyte proliferation and differentiation and cardiac development [24]. Meanwhile, *CUX1* is a cell-cycle regulator that plays an important role in the development of several tissues. In addition, it has been shown that *CUX1* can affect the regulation of the hair growth cycle in mice, while loss of its exons leads to hair curvature [25,26]. To explore the function of *CUX1* in mice, Luong and Ellis mutated different C-terminal positions of *CUX1*. Mutant mice exhibited retarded growth and had abnormal hair development. This indicates that *CUX1* plays an important role in hair follicle growth and development [6,19]. To verify the specific role of *CUX1* in DPCs, we analyzed whether *CUX1* can regulate the proliferation of DPCs by overexpressing and knocking down *CUX1*. Overexpression of *CUX1* promoted mRNA and protein expression of related proliferative genes, while the opposite observations were made after *CUX1* knockdown. The proliferation status and cell-cycle progression of DPCs were detected by CCK8, cell-cycle, and EdU assays, and it was found that overexpression of *CUX1* promoted the proliferation of DPCs, while knockdown of *CUX1* inhibited the proliferation of DPCs. These results suggest that *CUX1* can promote the proliferation of DPCs.

The Wnt/β-catenin signaling pathway plays a critical role in hair growth and development. It was found that *CUX1* plays its important regulatory role by affecting the Wnt/β-catenin signaling pathway. *CUX1* can regulate β-catenin expression and activate the Wnt/β-catenin signaling pathway in gliomas, and, when *CUX1* is knocked down, the expression of β-catenin at the mRNA and protein levels is suppressed. In addition, a TOP/FOP assay showed that downregulation of *CUX1* significantly inhibited the activity of the Wnt/β-catenin signaling pathway [27]. To investigate whether *CUX1* can regulate Wnt/β-catenin to exert its biological function in DPCs, we overexpressed and knocked down *CUX1* and then detected the mRNA expression levels of *WNT10*, *MMP7*, *C-JUN*, *PPARD*, *CCND1*, *FOSL1*, and *CTNNB1*. *CUX1* upregulated key Wnt/β-catenin genes, thus regulating the proliferation of DPCs.

## 5. Conclusions

In conclusion, we showed that *CUX1* can affect the expression of the cell proliferation genes *CDK2*, *PCNA*, and *CYCLIN D1* to some extent, promote the proliferation of DPCs and regulate the expression of key genes in the Wnt/β-catenin signaling pathway. The present study provides a basis for the elucidation of the molecular mechanisms underlying lambskin pattern formation.

## Figures and Tables

**Table 1:** Primer information.

Group	Forward Primer (5′-3′)	Reverse Primer (5′-3′)
siRNA-166	CCGCAACAGUAUUGGCAAATT	UUUGCCAAUACUGUUGCGGTT
siRNA-232	GCCGUGAGUUCAAGAAGAATT	UUCUUCUUGAACUCACGGCTT
siRNA-1870	GCCUGAGCCCAUGGGACAATT	UUGUCCCAUGGGCUCAGGCTT
siRNA-NC	UUCUCCGAACGUGUCACGUTT	ACGUGACACGUUCGGAGAATT

**Table 2:** Primer information.

Gene	Sequences (5′→3′)	Product Length
*CUX1*	F:GCACGACATTGAGACGGAG	160
	R:AGCTATGGTCTCAGCCTGGT	
*CDK2*	F:AGAAGTGGCTGCATCACAAG	92
	R:TCTCAGAATCTCCAGGGAATAG	
*PCNA*	F:CGAGGGCTTCGACACTTAC	97
	R:GTCTTCATTGCCAGCACATT	
*CYCLIN D1*	F:CCGAGGAGAACAAGCAGATC	91
	R:GAGGGTGGGTTGGAAATG	
*KRT71*	F:GGCTCATCCAGAGAATCCGC	102
	R:GAGCATTGTCACCCCTCTGT	
*CTNNB1*	F:CGCCTTCACTACGGACTACC	173
	R:GCACGAACCAGCAACTGAAC	
*WNT10B*	F:TCTCCTGTTCCTGGCGTTGT	101
	R:AGACTGTGTTGGCGGTCAG	
*C-JUN*	F:GCTTCCAAGTGCCGGAAAAG	184
	R:GCTGCGTTAGCATGAGTTGG	
*PPARD*	F:CTGCTCCTCACTCTCACTGC	233
	R:GGCACTTGTTGCGGTTCTTC	
*MMP7*	F:TGTGGAGTACCGGATGTTGC	166
	R:GTGGGATCACTTCGCTCCAT	
*FOSL1*	F:TGGTTCAGCCTCACTTCCTG	235
	R:TCGGTCAGTTCTTTCCTCCG	
*CCND1*	F:GCCGAGGAGAACAAGCAGAT	176
	R:GCGGTGATAGGAGAGGAAGC	
*GAPDH*	F:TCTCAAGGGCATTCTAGGCTAC	151
	R:GCCGAATTCATTGTCGTACCAG	

**Table 3:** Primary and secondary antibodies.

Antibody name	Purpose	Source	Dilution	Company
GAPDH	Primary antibody	Mouse	1:2500	Proteintech
CUX1	Primary antibody	Rabbit	1:2500	Proteintech
CDK2	Primary antibody	Rabbit	1:2500	Proteintech
PCNA	Primary antibody	Rabbit	1:2500	Proteintech
HRP goat anti-rabbit	Secondary antibody	Rabbit	1:3000	ABclonal
HRP goat anti-mouse	Secondary antibody	Mouse	1:3000	ABclonal

## Data Availability

Not applicable.
